# A mm-Sized Free-Floating Wireless Implantable Opto-Electro Stimulation Device

**DOI:** 10.3390/mi11060621

**Published:** 2020-06-25

**Authors:** Yaoyao Jia, Yan Gong, Arthur Weber, Wen Li, Maysam Ghovanloo

**Affiliations:** 1 Department of Electrical and Computer Engineering, North Carolina State University, Raleigh, NC 27606, USA; 2 Department of Electrical and Computer Engineering, Michigan State University, East Lansing, MI 48824, USA; gongyan@msu.edu (Y.G.); wenli@msu.edu (W.L.); 3 Department of Physiology, Michigan State University, East Lansing, MI 48824, USA; weberar@msu.edu; 4 Bionic Sciences Inc., Atlanta, GA 30316, USA; mghovan@ieee.org

**Keywords:** free-floating implants, inductive link, switched-capacitor-based optical/electrical stimulation, charge balancing

## Abstract

Towards a distributed neural interface, consisting of multiple miniaturized implants, for interfacing with large-scale neuronal ensembles over large brain areas, this paper presents a mm-sized free-floating wirelessly-powered implantable opto-electro stimulation (FF-WIOS2) device equipped with 16-ch optical and 4-ch electrical stimulation for reconfigurable neuromodulation. The FF-WIOS2 is wirelessly powered and controlled through a 3-coil inductive link at 60 MHz. The FF-WIOS2 receives stimulation parameters via on-off keying (OOK) while sending its rectified voltage information to an external headstage for closed-loop power control (CLPC) via load-shift-keying (LSK). The FF-WIOS2 system-on-chip (SoC), fabricated in a 0.35-µm standard CMOS process, employs switched-capacitor-based stimulation (SCS) architecture to provide large instantaneous current needed for surpassing the optical stimulation threshold. The SCS charger charges an off-chip capacitor up to 5 V at 37% efficiency. At the onset of stimulation, the capacitor delivers charge with peak current in 1.7–12 mA range to a micro-LED (µLED) array for optical stimulation or 100–700 μA range to a micro-electrode array (MEA) for biphasic electrical stimulation. Active and passive charge balancing circuits are activated in electrical stimulation mode to ensure stimulation safety. In vivo experiments conducted on three anesthetized rats verified the efficacy of the two stimulation mechanisms. The proposed FF-WIOS2 is potentially a reconfigurable tool for performing untethered neuromodulation.

## 1. Introduction

Neurological disorders, such as Alzheimer’s disease, Epilepsy, Multiple sclerosis, Parkinson’s disease, and Stroke, are increasingly recognized as major causes of disability and death worldwide as societies grow older [1]. In many cases, these diseases cannot be treated adequately by medication alone [1]. Therefore, new therapies are required in addition to the traditional pharmaceutical treatment. This is where new neuro-modulation based therapies are considered [2,3]. Electrical stimulation is a well-established neuromodulation strategy, which modulates neural activities by injecting charge into target neural tissue using implantable microelectronic devices (IMD) [4]. Various IMDs with electrical stimulation capability have been developed and used clinically since the 60′s not only for neurological disorders but also for sensory deficits, such as cochlear implants [5,6]. Optical stimulation is a much more recent approach with distinct advantages of cell-type specificity, high temporal precision, and rapid reversibility, which has become quite popular for neuromodulation in research settings [7,8]. Optical stimulation, however, is power-hungry and not practical with primary sources of energy in today’s IMDs. There are significant ongoing efforts on the development of neural interfaces with optical stimulation capability towards clinically viable devices [9,10,11,12]. Therefore, it would be promising to have a neural interface device equipped with both optical and electrical stimulation capabilities offering the best of both neuromodulation methods and offering researchers ultimate flexibility in executing advanced neuromodulation paradigms.

For the in vivo assessment of the IMDs, cost-effective animal models, such as rodents, are often used [2,3]. The conventional structure of the IMDs is that a central control unit connects to multiple recording and/or stimulation sites, each of which has limited coverage area around the electrode [13,14]. Recent studies have suggested that neural functions in the brain always result from the synergy of a large distributed network of neurons [15,16]. As the number of recording/stimulation channels increases for large brain area coverage, the conventional IMD structure will face several scalability issues. The entire IMD will become very bulky. The heat generation will be concentrated around the central control unit, challenging the safety limitation of the tissue in terms of electromagnetic absorption and heat generation. The IMD will also suffer from the high risk of the failure due to the massive interconnects [13,14]. A novel architecture, in which a large number of tiny IMDs distribute over a large brain area, could be a viable solution for the abovementioned scalability issues [17]. Thanks to the small footprint (mm-sized), the tiny IMDs will cause less damage to the surrounding tissue [18].

Recently, a few tiny neural interface IMDs have been presented for neuromodulation with an impressive reduction in the device size [19,20,21,22,23,24,25,26,27]. In [20], a 2.2 mm^3^ stimulator supports a single channel (1-ch) electrical stimulation via ultrasound power harvesting. A microelectrical stimulator, 0.009 mm^3^ in volume, is presented in [21] for 1-ch electrical stimulation. In [22,23,24,25], multiple optical stimulators assembled by different energy-harvesting components, e.g., a coil, a stretchable antenna, or a photovoltaic cell array, are designed for 1-ch optical stimulation. The abovementioned tiny implants only support a single type of stimulation with a single channel. In [26], an inductively-power device is equipped with electrical and optical stimulation capabilities, but its bulky volume makes it hard to be implanted. The implant presented in [27] has both electrical and optical stimulation functions implemented in a mm-sized implant, but the number of channel-count for each type of stimulation is low. Generally speaking, the main constrains for the tiny stimulation implants are device size, power budget, and flexibility in function. New architectures should be explored to reach the best compromise among the conflicting space, power, and functionality tradeoffs.

In this paper, we are demonstrating a new mm-sized free-floating wirelessly-powered implantable opto-electro stimulation (FF-WIOS2) device capable of 16-ch optical stimulation and 4-ch electrical stimulation. The FF-WIOS2 device, built upon our previous work in [28], is the most versatile mm-sized neuromodulation device to the best of our knowledge. The performance of the entire system was also successfully validated in vivo on anesthetized rats by observing evoked local field potentials (LFPs) under each stimulation mode and immunostained tissue response. Section 2 describes the system overview, the FF-WIOS2 system-on-chip (SoC) design, system implementation, and in vivo experiment design. Section 3 presents the benchtop measurement results and in vivo experiment results. Section 4 discusses the advantages and limitations of the system compared to the state-of-the-arts and the future work, followed by concluding remarks.

## 2. Materials and Methods

### 2.1. System Overview

**Figure 1:**
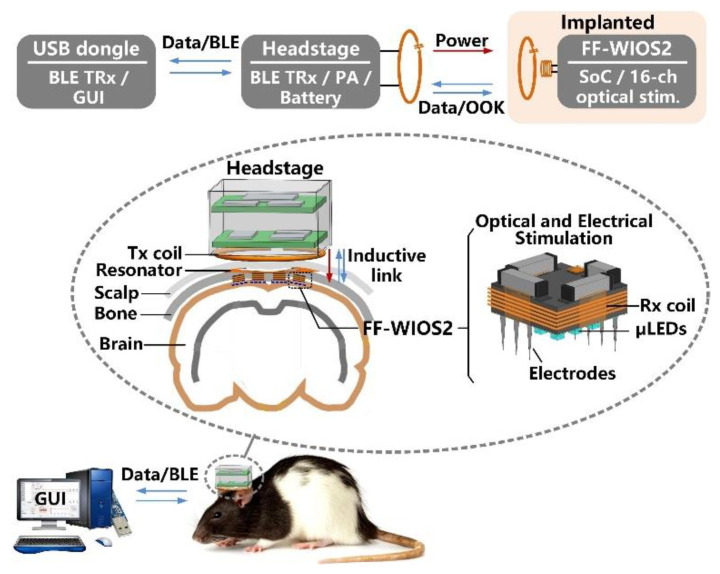
Conceptual view of the system setup for operating multiple mm-sized free-floating wirelessly-powered implantable opto-electro stimulation (FF-WIOS2) devices, distributed on a freely moving rat brain.

Figure 1 shows the conceptual system setup of wirelessly operating the distributed array of FF-WIOS2 devices on the brain surface of a freely behaving rat. A power amplifier (PA) headstage delivers power to the FF-WIOS2 devices through a 3-coil inductive link operating at 60 MHz. The 3-coil inductive link includes (1) a transmitter (Tx) coil, *L*_Tx_, located at the bottom of the headstage, (2) a resonator, *L*_Res_, implanted under the scalp but above the skull, and (3) a Rx coil, *L*_Rx_, wired-wound around the FF-WIOS2 SoC. The purpose of adding *L*_Res_ is to increase the magnetic field intensity within the area that is encompassed by *L*_Res_. The 3-coil inductive link, together with the Bluetooth Low Energy (BLE) link built between the headstage and the external USB dongle, support wireless data communication with the FF-WIOS2 devices. In the downlink data transmission, stimulation parameters, set in the graphical user interface (GUI), are sent to the headstage via the BLE link before being delivered to the FF-WIOS2 device by on-off-keying (OOK) the power carrier. In the uplink data transmission, the voltage output of the doubler, which reflects the amount of power delivery, is transferred to the headstage by load-shift-keying (LSK) for closed-loop power control (CLPC) and eventually sent to the PC via BLE for real-time display.

The FF-WIOS2 device implementation offers the possibility of both electrical and optical stimulation using the same device, even though not at the same time. Users can activate dedicated stimulation sites for electrical and optical stimulation, wirelessly from outside. The FF-WIOS2 device employs a polyimide film as a substrate, which carries four off-chip surface mount device (SMD) capacitors on the top side, and micro-LEDs (µLEDs) for optical stimulation and microelectrodes for electrical stimulation on the bottom side. The silicon die is fixated in the top center of the polyimide film, followed by wire bonding to form electrical connections between the silicon die and the polyimide film. Details of the assembly procedure are similar to what is described in [28].

### 2.2. FF-WIOS2 SoC Architecture

**Figure 2:**
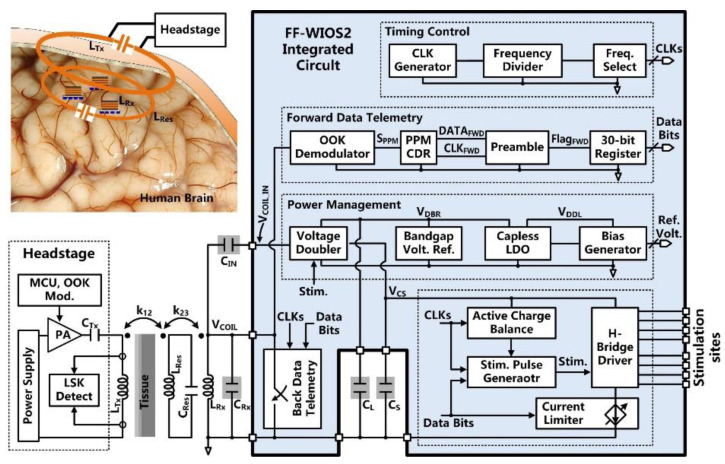
Block diagram of the FF-WIOS2 system-on-chip (SoC) architecture.

The FF-WIOS2 SoC block diagram is shown in Figure 2. A key challenge in the FF-WIOS2 SoC design is how to utilize the limited amount of power received by the FF-WIOS2 device to apply effective neuromodulation. For the power-hungry optical stimulation, instantaneous output power to the selected µLED needs to be large enough for the intensity of the resulting flash of light to surpass the optogenetic neuromodulation threshold [29]. Thus, we utilized a switched-capacitor-based stimulation (SCS) architecture [30] in the FF-WIOS2 SoC.

Once stimulation parameters are received, and stimulation patterns are set, the FF-WIOS2 SoC starts the sequence of charging, delay, and stimulation in a loop while monitoring and reporting back the amount of received power for the CLPC. This sequence is interrupted only if the stimulation parameters need to be updated. More specifically, a storage capacitor, *C*_S_, is periodically charged by the charger embedded in the voltage doubler. The target charging voltage is set by the back data telemetry circuit. At the onset of stimulation, *C*_S_ is detached from the charger and dumps its charge into either a designated µLED for optical stimulation or a pair of electrodes for electrical stimulation, through four multiplexers (MUX) in an H-bridge. The upper bound of the stimulation current is set by the current limiter. The stimulation pulses are adjustable in terms of peak current amplitude, pulse width, and frequency. During each stimulation pulse, the active charge balancing (CB) circuit will dynamically adjust the duration of the anodic phase to neutralize the amount of charge dumped into the tissue during the cathodic phase. After each stimulation pulse, the passive CB will short the selected pair of electrodes to the ground to eliminate any residual charge.

In the power management block, the AC voltage across the *L*_Rx_*C*_Rx_-tank, *V*_COIL_, is converted to a DC voltage, *V*_DBR_, by the voltage doubler. Taking the bandgap reference voltage, *V*_BGR_, as input, the capacitor-less low dropout (LDO) regulator further stabilizes *V*_DBR_ to generate a system supply voltage, *V*_DDL_. In the forward data telemetry, the OOK-modulated *V*_COIL_, carrying the stimulation parameter information, is first converted to pulse-position-modulated (PPM) signal, *S*_PPM_, by the OOK demodulator. Then, *S*_PPM_ is converted to the synchronized data, *DATA*_FWD_, and clock, *CLK*_FWD_, by the pulse-position-modulated clock-data recovery (PPM-CDR) circuit for setting the stimulation parameters. The back telemetry block, which modulates the power carrier by switching the Rx coil, is part of the CLPC for maintaining *V*_DBR_ at 4.2 V.

**Figure 3:**
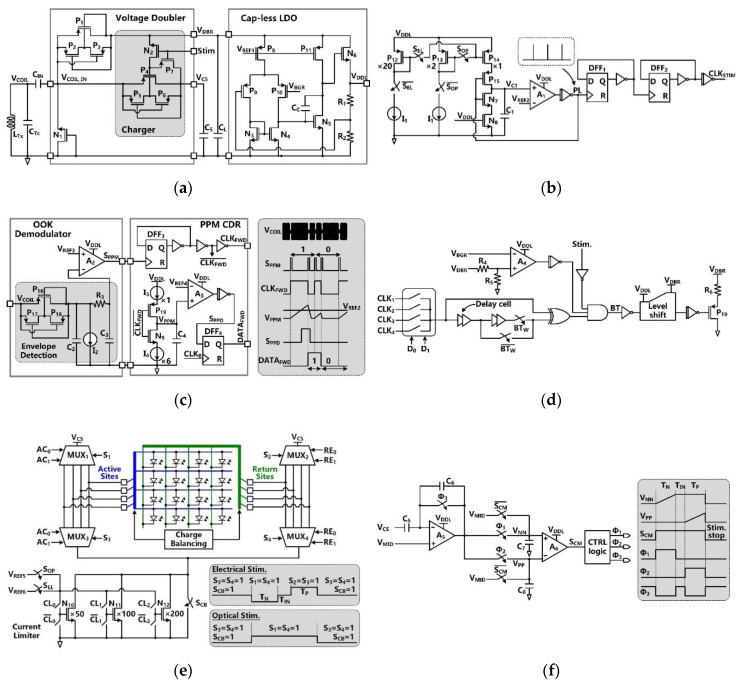
Schematic diagram of (**a**) the voltage doubler with built-in charger and the cap-less LDO, (**b**) the clock generator for the timing of stimulation and charging functions, (**c**) the OOK demodulator and PPM-CDR in forward data telemetry, (**d**) the LSK back telemetry, (**e**) the stimulation driver in H-bridge configuration, and (**f**) the active charge balancing (CB) circuit.

*V*_COIL_ is rectified by the voltage doubler and further regulated by the capless LDO, resulting in the SoC supply voltage of *V*_DDL_ =1.8 V, as shown in Figure 3a. The built-in charger of the voltage doubler is part of the SCS architecture and takes care of charging *C*_S_ [30]. This built-in charger is under control of a stimulation signal, *Stim*. During stimulation, *Stim* = ‘1′, the charger is disabled to avoid *V*_DDL_ drop, while *C*_S_ is connected to the stimulation sites and dumps its stored charge into either a designated µLED or a pair of microelectrodes for optical or electrical stimulation, respectively.

In Figure 3b, the clock generator circuit provides a reference clock, *CLK*_STIM_, for charging and stimulation functions. This is an astable circuit, in which the buffered output signal, *PL*, of the comparator, *A*_1_, controls the charging and discharging of *C*_1_ via switches, *P*_15_ and *N*_7_. In the phase of *PL* = ‘0′, the switch *P*_15_ is on, and *C*_1_ is charged by either *P*_12_ or *P*_13_. Once the capacitor voltage, *V*_C1_, reaches *V*_REF*2*_, *PL* is set to ‘1′. The switch *N*_7_ is on, and *C*_1_ is discharged in a short period, generating a single narrow *PL* pulse, which is converted to *CLK*_REF_ with 50% duty cycle through a frequency divider. The *CLK*_REF_ frequency is decided by the charging current, which is controlled by *S*_EL_ and *S*_OP_.

Figure 3c shows the schematics of the OOK demodulator and PPM-CDR. In the OOK demodulator, the envelope of the OOK-modulated *V*_COIL_ is extracted and compared with *V*_REF3_ to provide *S*_PPM_. In the PPM-CDR block, *S*_PPM_ is converted to *CLK*_FWD_ using a frequency divider. *CLK*_FWD_ controls the timing and amplitude of *V*_PPM_ by alternately charging and discharging *C*_4_ through current sources, *I*_3_ and *I*_4_, respectively. If positioning ratio among three consecutive *S*_PPM_ pulses is 4:1, *I_3_* charges *C*_4_ for a longer time, and *V*_PPM_ exceeds *V*_REF4_ during *CLK*_FWD_ = ‘1′, leading to *S*_PPD_ = ‘1′. Then, *S*_PPD_ is sampled in DFF_4_, leading to *DATA*_FWD_ = ‘1′, while the recovered *DATA*_FWD_ is synchronized with *CLK*_FWD_ in the PPM-CDR. If the positioning ratio is 1:4, *V*_PPM_ cannot reach *V*_REF4_ during *CLK*_FWD_ = ‘1′, resulting in *DATA*_FWD_ = ‘0′.

LSK back telemetry is adopted for implementing a CLPC for the FF-WIOS2 device and sets the maximum *V*_DBR_ at 4.2 V. In Figure 3d, the timing of the back telemetry pulse, *BT*, is adjustable in the range of 160 Hz to 1 kHz by two control bits. The pulse width of *BT* (1 µs or 2 µs) is decided by the number of delay cells that are engaged in generating this pulse. Once the divided *V*_DBR_ exceeds *V*_BGR_, *BT* pulses are generated to short *L*_Rx_. Since the load of *L*_Rx_ is reduced, the voltage across *L*_Tx_ is increased correspondingly. When the stimulation pulses are active, the *BT* pulses are disabled to prevent interference.

The stimulus driver, shown in Figure 3e, employs four 4:1 multiplexers (MUXs) in an H-bridge configuration with a current limiter. To achieve a compact SoC design, the stimulus driver is used for both optical and electrical stimulation. The MUXs select a pair out of four active and four return sites by control signals *AC*_1_-*AC*_2_ and *RE*_1_-*RE*_2_, respectively. In optical stimulation mode, current flows only in one direction by MUX_1_-MUX_4_ group. In electrical stimulation mode, however, both the MUX_1_-MUX_4_ pair and the MUX_2_-MUX_3_ pair are utilized alternatively to generate anodic and cathodic stimulation phases in opposite directions. Either a µLED or a pair of microelectrodes is selected out from the row-column format. The current limiter consists of a 3-bit programmable current sink with binary-weighted transistors. The transistors can be selectively biased at two reference voltages, providing different current ranges needed for optical and electrical stimulation. The switch, *S*_CB_, is utilized for passive CB after stimulation.

The active CB circuit, which is designed to ensure charge-balanced biphasic stimulation, is shown in Figure 3f. In the active CB circuit, a capacitive-feedback amplifier integrates the discharged voltage of the storage capacitor, *V*_CS_, to detect the amount of negative and positive charge injected into the tissue. Before stimulation, the charge monitoring signal, *S*_CM_, stays at ‘0′, and the amplifiers *A*_1_ and *A*_2_ operate as buffers, which are biased at the half supply voltage, *V*_MID_. When the stimulation starts, *S*_CM_ jumps to ‘1′. During the cathodic phase with a predefined duration of *T*_N_, *A*_1_ becomes a capacitive-feedback amplifier, and *A*_2_ operates as a comparator. The storage capacitor, *C*_S_, is alternately connected to the selected active and return stimulation sites to supply stimulus current. The sensing voltage across *C*_7_, *V*_NN_, linearly increases as *V*_CS_ decreases during *T*_N_, and stays at its final value until the end of the stimulation pulse. During the intermediate delay period, *T*_IN_, *A*_1_ operates as a buffer to reset its output voltage at *V*_MID_. When *C*_S_ discharges during the anodic phase, *T*_P_, the sensing voltage across *C*_8_, *V*_PP_, increases. When the amounts of *V*_PP_ and *V*_NN_ increments are equal, *S*_CM_ = ‘0′ again, and the positive stimulation stops to ensure that the net injected and withdrawn charges are zero.

### 2.3. System Implementation

**Figure 4:**
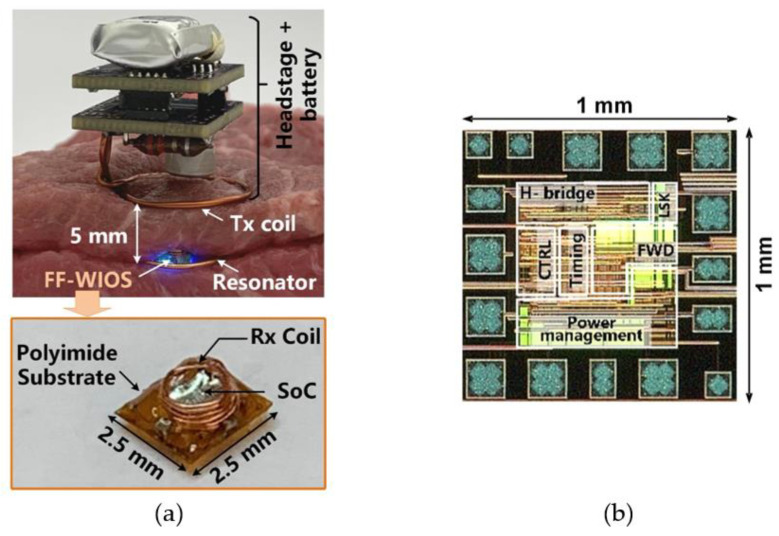
(**a**) In vitro system setup with a close-up view of the FF-WIOS2 device. (**b**) The fabricated FF-WIOS2 SoC micrograph.

Figure 4a shows the in vitro test setup using tissue layers for preliminary evaluation of the FF-WIOS2 system operation. The headstage, consisting of two stacked PCBs and *L*_Tx_, is powered by a 3.7 V, 280 mAh rechargeable LiPo battery, resulting in 15 × 15 × 23 mm^3^ in size and weight of 4.2 g. The headstage is placed face down above the tissue so that *L*_Tx_ can be close to *L*_Res_ and *L*_Rx_. Similar to the conceptual view of the system implementation in Figure 1, the assembled FF-WIOS2 device and *L*_Res_ are in approximately the same plane, and 5 mm below *L*_Tx_. *L*_Tx_, *L*_Res_, and *L*_Rx_ are concentrically aligned in this test. The design and optimization of the 3-coil WPT link have been described in [28]. The FF-WIOS2 device is built following the assembly process described in [28], resulting in device dimensions of 2.5×2.5×1.5 mm^3^ and weight of 15 mg. Figure 4b shows the FF-WIOS2 SoC, which was fabricated in the TSMC 0.35-μm 4M2P standard CMOS process, occupying 1 × 1 mm^2^ of silicon area including pads.

**Figure 5:**
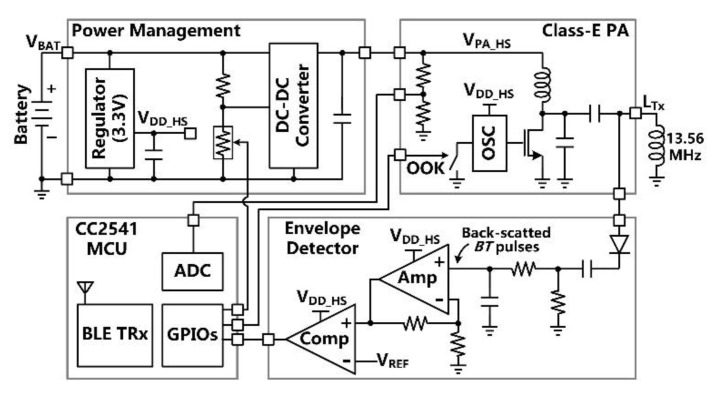
Block diagram of the headstage.

Figure 5 shows the simplified schematic diagram of the headstage which is made of commercial off-the-shelf (COTS) components. The main tasks of the headstage include (1) delivering sufficient power via the 3-coil inductive link to the FF-WIOS2 device, which drives a selected µLED in a user-defined pattern, (2) establishing BLE link with the nearby PC, (3) transferring stimulation parameters to the FF-WIOS2 device, and (4) recovering *BT* pulses for the CLPC. The DC-DC converter directly powers the Class-E PA with the supply voltage, *V*_PA-HS_, adjustable from 4 V to 8 V, while the linear regulator powers the rest circuits with the supply voltage *V*_DD-HS_ = 3.3 V. The Class-E PA, which a 13.56 MHz oscillator drives the power MOSFET, delivers *L*_Tx_. The CC2541 microcontroller unit (MCU) in the headstage OOK modulates the PA’s power carrier by on/off switching the oscillator for downlink data transmission. In the envelope detector, the voltage across *L*_Tx_ is first filtered and amplified to extract the voltage variation across *L*_Tx_. Then, the extracted voltage variation is compared with a reference voltage, *V*_REF-HS_, to recover *BT* pulses, which are monitored by the headstage MCU in real-time.

Given that the FF-WIOS2 device has limited power budget and size, the rule of thumb in CLPC implementation is to reduce the complexity and power consumption of the FF-WIOS2 device, often at the cost of more complexity on the headstage side, which is relatively less constrained. Similar to [31], when *BT* pulses are detected, the CLPC algorithm controls the headstage MCU to decrease *V*_PA-HS_; otherwise, *V*_PA-HS_ is continually increased by default at an adjustable rate. In steady-state, *V*_PA-HS_ maintains within a certain range, resulting in stabilized power delivered to the FF-WIOS2 device. The divided *V*_PA-HS_ is also sampled by the built-in analog-to-digital converter (ADC) of the headstage MCU and sent out via BLE to the PC for real-time display in the GUI.

### 2.4. In Vivo Experiment Design

We conducted in vivo experiments to verify the efficacy of the FF-WIOS2 SoC by evoking neural activities in the primary visual cortex (V1) of anesthetized rats. We conducted animal tests on three male adult rats (Sprague Dawley, 350–400 g) by following our established protocols approved by the Institutional Animal Care and Use Committee (IACUC) at Michigan State University. We injected adeno-associated virus (AAV) that carries optogenetics opsin (AAV-hSyn-hChR2 (H134R)-mCherry; UNC Vector Core) bilaterally into each subject’s V1 using the stereotaxic surgery protocol in [9]. After virus injection, the rats were housed in the animal facilities for around 4 weeks until the cortical neurons expressed light excitability in channelrhodopsin-2 (ChR2).

**Figure 6:**
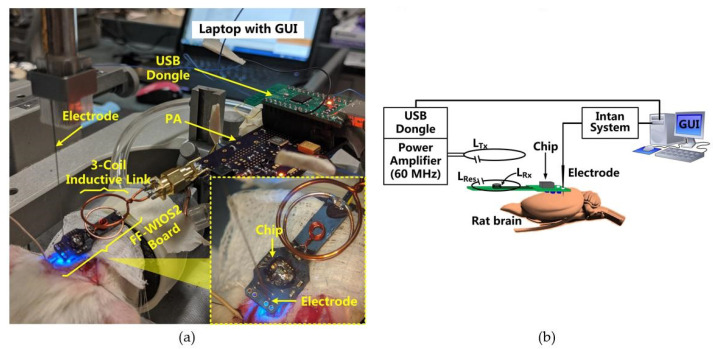
(**a**) In vivo experimental setup with its (**b**) block diagram.

Figure 6 shows the in vivo experiment setup when optical stimulation was conducted on the rat’s V1 using a FF-WIOS2 prototype board, which is designed specifically for acute animal studies. The 25 × 9 mm^2^ board includes the FF-WIOS2 SoC, *L*_Rx_, *L*_Res_, and off-chip capacitors assembled on the top side as well as a 2 × 2 µLED array (0.5 × 1 × 0.4 mm^3^, LBQH9G, OSRAM) assembled on the bottom side. The FF-WIOS2 evaluation board was placed over the skull of the rats with two µLEDs aligned with each side of the V1 lobe.

In this setup, a class-E PA, made of COTS components, wirelessly delivers power to the FF-WIOS2 SoC through an optimized 60 MHz 3-coil inductive link [28]. Once the USB dongle receives stimulation parameters set in GUI, it OOK modulates the power carrier of the PA to wirelessly deliver the stimulation parameters to the FF-WIOS2 SoC. In return, the FF-WIOS2 SoC wirelessly transfers its supply voltage information to the USB dongle for CLPC via LSK modulation of the power carrier (back telemetry). In this setup, the 60 MHz PA board is a substitute for the headstage shown in Figure 1. Instead of building the BLE link between the USB dongle and the headstage in Figure 1, the USB dongle is directly connected to the PA board to simplify the in vivo experiment setup.

We applied unilateral stimulation with user-defined stimulation mode and parameters on the left V1 of the animal subject under anesthesia. We simultaneously recorded local field potentials (LFPs) through a tungsten electrode which penetrated into the left V1 cortical layers at a depth of 100 µm. We used a commercial 32-ch Intan system (RHD2132, Intan Technologies, Los Angeles, CA, USA) to amplify and digitize the recorded LFP signals and then uploaded the digitized LFP data to the PC for post data analysis using a MATLAB Chronux toolbox (Version 2.12, MathWorks, Natick, MA, USA).

In optical stimulation mode, the selected µLED was controlled by stimulation pulses under current limits of 12 mA and 1.7 mA, corresponding to the light intensity of 11.8 mW/mm^2^ and 1.5 mW/mm^2^, respectively. Each optical stimulation started with 6.4 ms light exposure and lasted 1 s. Electrical stimulation under current limits of 100 µA and 700 µA were also applied via tungsten electrodes. The stimulation pulses with a predefined negative pulse width of 350 µs and an interval delay of 100 µs were applied every 50 ms. In each stimulation mode, the LPF was recorded at different stimulation strengths to be later compared. We expected to observe higher LFPs variations with higher stimulation amplitude. In addition, since neuronal oscillation is a fundamental component of brain function and plays an important role in large-scale neuronal computations [32,33], we expected to observe neuronal oscillations in rat’s V1 caused by the stimulation effects.

## 3. Results

### 3.1. Benchtop Measurement Results

**Figure 7:**
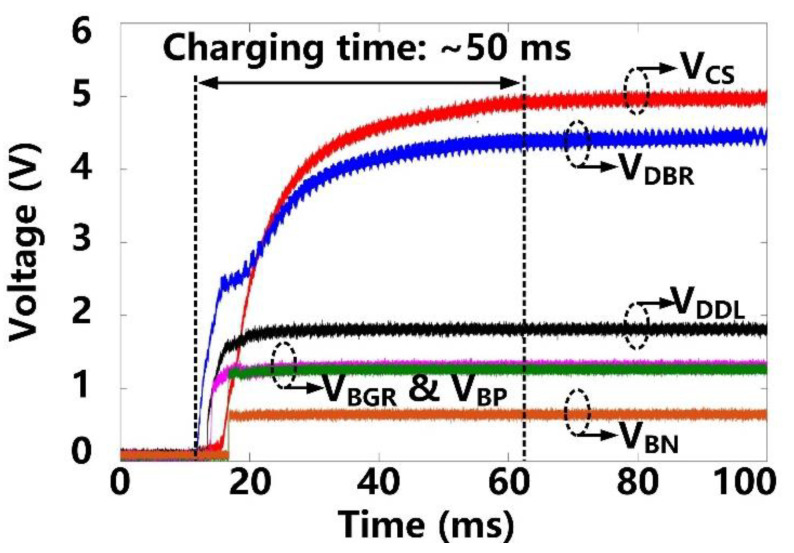
Transient waveforms of the power management block at starting up.

**Figure 8:**
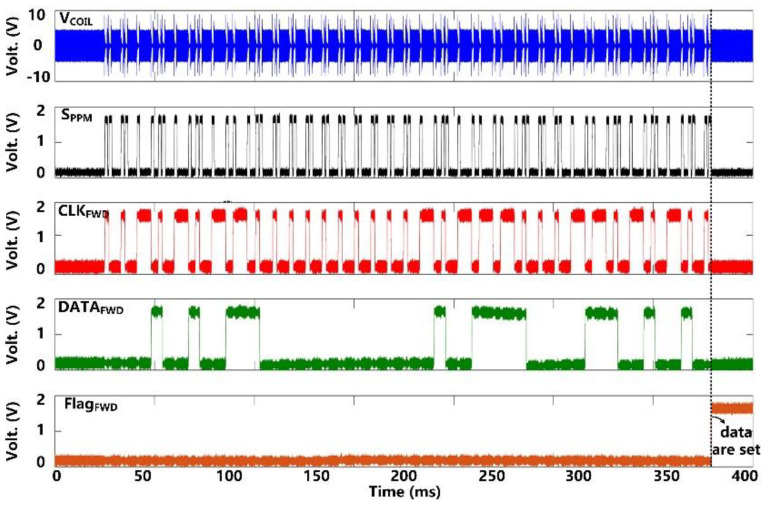
Measured results of forward data telemetry.

Following startup, it takes ~50 ms for *V*_CS_ and *V*_DBR_ to stabilize at their steady-state target voltages of 5 V and 4.2 V, respectively, as shown in Figure 7. *V*_DDL_ and *V*_BGR_ have already been stabilized at 1.8 V and 1.2 V, respectively, at this point, and so do, *V*_BP_ and *V*_BN_ references at 1.2 V and 0.6 V, respectively. Figure 8 shows the measurement results of the forward data telemetry. *V*_COIL_ is OOK-demodulated to generate *S*_PPM_, which is converted to synchronized 50 kbps *CLK*_FWD_ and *DATA*_FWD_ by PPM-CDR. *S*_PPM_ with a pulse position ratio of 4:1 generates *DATA*_FWD_ = ‘1′. On the contrary, when the positioning ratio is 1:4, *DATA*_FWD_ = ‘0′. Once the pre/post-amble data bits are matched with a predefined 10-bit value, a flag, *Flag*_FWD_, raises, and received data bits are saved in registers to set the stimulation parameters.

**Figure 9:**
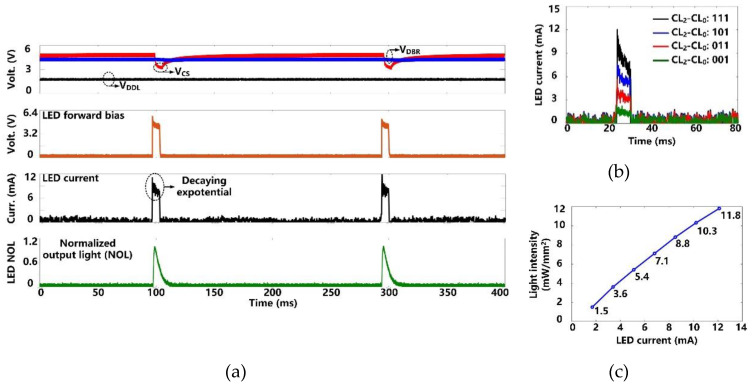
(**a**) Measured optical stimulation waveforms. (**b**) Measured µLED current at different stimulation current settings. (**c**) Measured light intensity as a function of the μLED current.

Figure 9a shows the charging and discharging of the *C*_S_ = 10 µF to generate optical stimulation with the settings, pulse width of 6.4 ms, frequency of 5 Hz, and current limit of 12 mA. Once stimulation starts, *C*_S_ dumps its charge into the target µLED, resulting in a decaying exponential current waveform with a drop of ~2.5 V in *V*_CS_. Since *V*_DBR_ remains above the minimum level of 2.6 V, *V*_DDL_ is not unaffected during the *C*_S_ discharging. We used the photodetector (Newport 883-SL, Newport Corporation, Irvine, CA, USA) of an optical power meter (Newport 1835-C, Newport Corporation, Irvine, CA, USA) to collect the emitted light from the µLED (0.5 × 1 × 0.4 mm^3^, LB QH9G, OSRAM, Munich, Germany) during stimulation pulse. As expected, the normalized output light (NOL) can repeat the stimulation current variation shown in the µLED datasheet [34]. After each stimulation, *C*_S_ is recharged to the target voltage within 30 ms. This charging speed allows a maximum stimulation rate of 10 Hz.

We measured the µLED current under four current settings (*CL*_0_-*CL*_2_) by measuring the voltage across a 10 Ω current-sensing resistor in series with the µLED. In Figure 9b, the peak value of the µLED current increases from 1.7 mA to 12 mA in a step of 3.4 mA. The light intensity at each current level was also measured. The measurement results are well-matched with the µLED datasheet [34]. As shown in Figure 9c, the resulting light intensity is from 1.5 mW/mm^2^ to 11.8 mW/mm^2^, which is above the estimated 1 mW/mm^2^ threshold for effective optogenetic neuromodulation [29].

**Figure 10:**
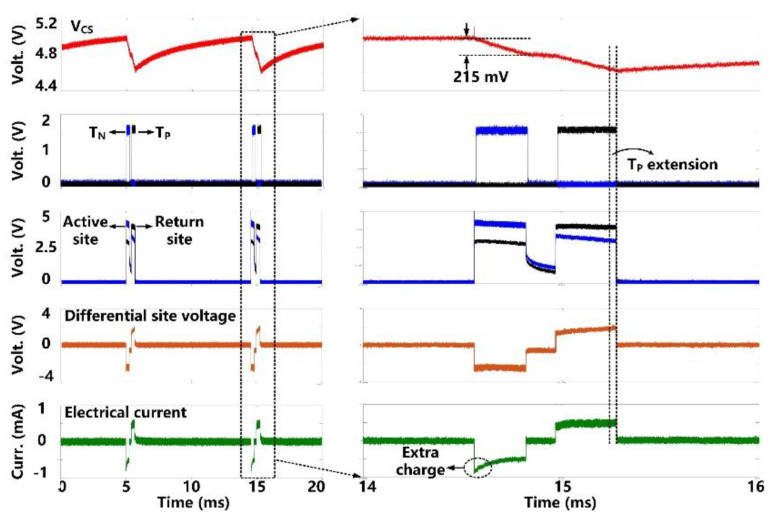
Measured electrical stimulation waveforms with active charge balancing.

The charge-balanced electrical stimulation waveforms are presented in Figure 10. The storage capacitor (*C*_S_ = 1 µF) delivers cathodic and anodic charge stimuli to a tissue model consisting of *R*_S_ = 2 kΩ and *C*_DL_ = 500 nF in series [30], with current amplitude limited to ±700 μA. We set the duration of the cathodic phase, *T*_N_, to 350 μs, resulting in the *V*_CS_ decrease of 215 mV. Since the stimulation current during the anodic phase, *T*_P_, drops a little bit, we can see that the active CB circuit dynamically extends the anodic phase duration to 420 μs until *V*_CS_ has the same voltage drop during the two phases. As a result, the amount of injected and withdrawn charges can be neutralized. For additional charge balancing, the passive CB circuit shorts the stimulation sites to the ground after each stimulation for a predefined period of 100 μs.

**Figure 11:**
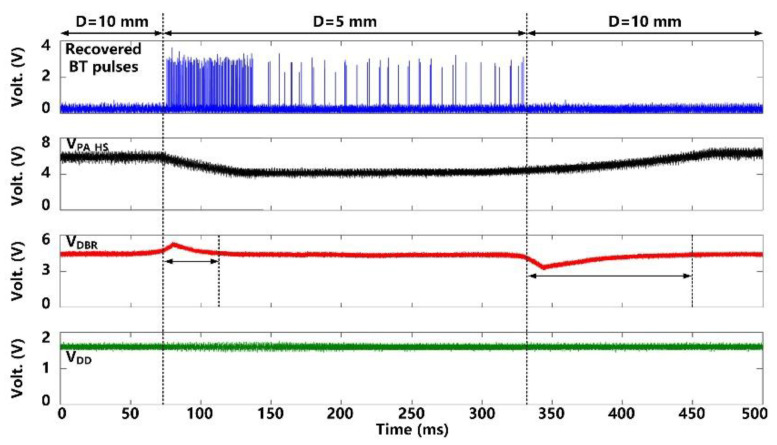
Measured waveforms under CLPC operation, when moving the headstage to change the distance between *L*_Tx_ and *L*_Res_, *D*.

Figure 11 shows the CLPC operation when the headstage is moved manually to change the distance, *D*, between *L*_Tx_ and *L*_Res_ from *D* = 10 mm to 5 mm and then back to 10 mm. As the headstage gets closer to the FF-WIOS2 device, *V*_DBR_ becomes larger than a certain threshold (4.2 V), indicating that there is more than enough power available to the FF-WIOS2 device. As a result, *BT* pulses are generated and recovered in the headstage. In response, the CLPC starts reducing *V*_PA-HS_ to compensate for this perturbation. It takes ~40 ms for *V*_DBR_ to return back to 4.2 V and *V*_PA-HS_ to settle in its new value. *V*_PA-HS_ drops from 6 V to 4 V, corresponding to the power consumption of the headstage reducing from 105 mW to 98 mW. In the new stable status, We can see that even in the stable status with *D* = 5 mm, *BT* pulses are still generated and covered as a result of the CLPC operation principle that *V*_PA-HS_ continually increases by default till the appearance of *BT* pulses again. As the headstage moves back to its original location, *V*_DBR_ drops. With the help of CLPC, *V*_DBR_ and *V*_PA-HS_ return back to 4.2 V and 6 V, respectively, after ~100 ms. It is important to note that during this significant disturbance, the SoC supply voltage, *V*_DDL_, always remains stable at 1.8 V.

**Figure 12:**
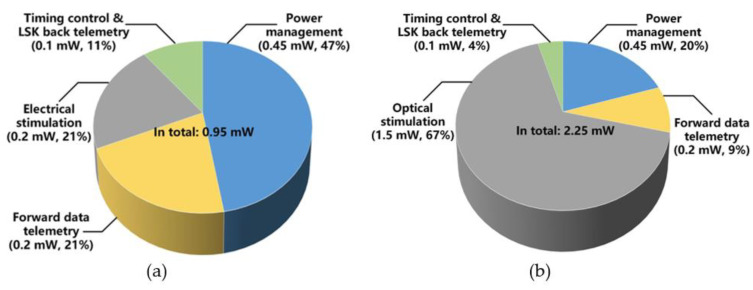
Power consumption of each block in the FF-WIOS2 device when applying (**a**) electrical stimulation and (**b**) optical stimulation.

The pie-charts in Figure 12 shows the average power consumption by the main FF-WIOS2 circuit blocks during electrical and optical stimulation. The total power consumption when electrical stimulation is set at its maximum amplitude is 0.95 mW. In this case, the power management block has the highest power consumption (47%), followed by the electrical stimulation (21%) and the forward data telemetry (21%) blocks. The average power consumption of the SoC with maximum optical stimulation is 2.25 mW, and according to Figure 12b, 67% of the total power is consumed on the optical stimulation function. Table 1 summarizes the measured specifications of the FF-WIOS2 SoC.

### 3.2. In Vivo Experiment Results

**Figure 13:**
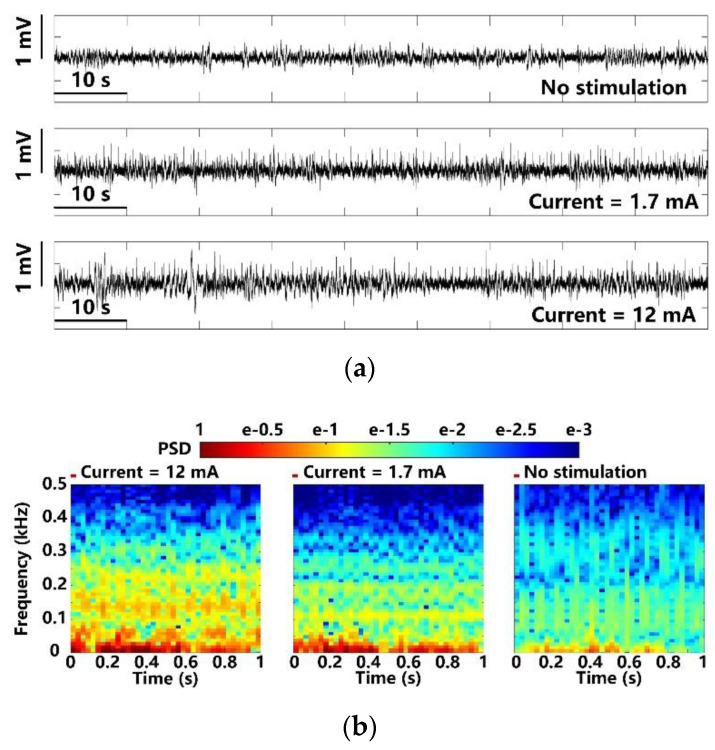
LFP analysis in terms of (**a**) amplitude variation and (**b**) normalized PSD with maximum and minimum optical stimulation.

In Figure 13a, the LPFs recorded at different optical stimulation strengths are compared over a time span of 90 s. Photoelectric artifacts induced from light stimulation are recorded with the LFPs [7,8]. The spontaneous LFPs in the upper trace are compared with light-evoked LFPs under weak and strong stimulation at 1.7 mA and 12 mA peak currents. It can be noted that the LFP variations under 12 mA stimulation current are significantly larger. The FFT transformation is applied to the averaged LFPs over 100 optical stimulations [9,35]. The resulting power spectral densities (PSD) are normalized and mapped onto a time-frequency graph, as shown in Figure 13b, where colors indicate the normalized PSD, and x and y axes indicate the 1 s stimulation period and 1–500 Hz LFP frequency range, respectively. The LFPs after the optical stimulation show significant increase in PSD, which is concentrated within a relatively narrow band of 1–100 Hz. The stimuli at 1.7 mA causes a slight increase in PSD as compared to the PSD under stimuli at 12 mA. Moreover, compared to the control, the light-evoked LFP recordings from the stimulated lobe show distinct PSD patterns, where recurrent low-frequency oscillations are observed.

**Figure 14:**
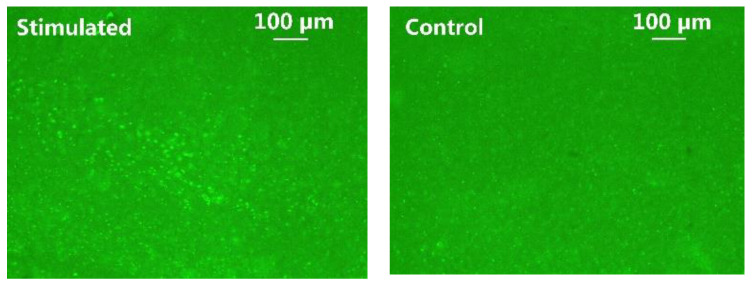
C-Fos expression in the left (stimulated) vs. right (control) V1 lobes.

C-Fos has been widely used as a biomarker to validate neuronal activity induced by optical stimulation [9,36]. It was performed to identify the increased expression of c-Fos in addition to LFP analysis. The animal subject received a 45-min optical stimulation on the left V1 lobe with 2 ms pulse width, 2.5 Hz pulse rate, and 10 mA stimulation current, while the intact right V1 lobe of the same animal acted as control. In Figure 14, the fluorescent images of the post-processed 50 µm-thickness brain slice were taken under 10× magnification. The green fluorescence spots indicate cells expressing c-Fos. Significantly higher c-Fos expression of ChR2 transfected cells is observed in the left V1 lobe, as opposed to the control side. These immunohistochemical analysis results further validate the efficacy of the optical stimulation.

**Figure 15:**
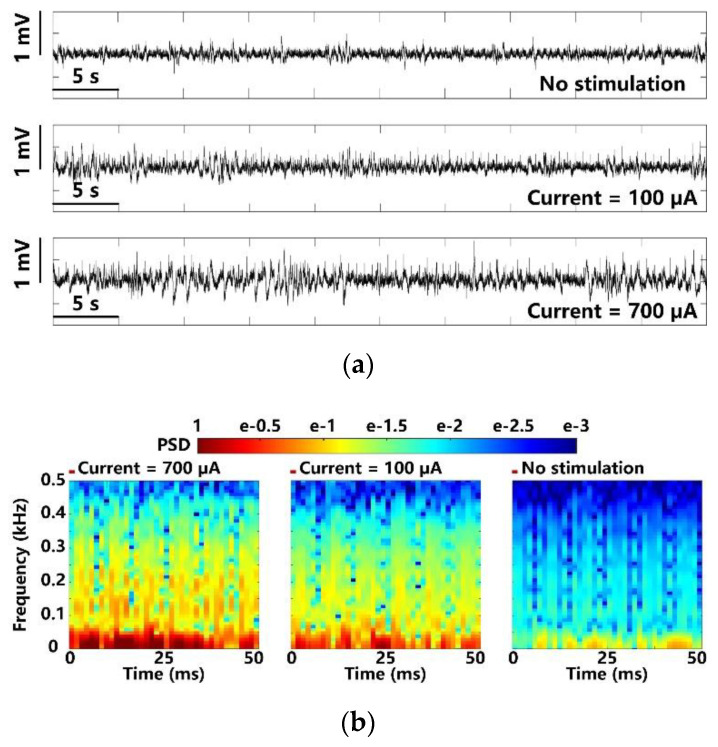
LFP analysis in terms of (**a**) amplitude variation and (**b**) normalized PSD with maximum and minimum electrical stimulation.

In addition to optical stimulation, the same experimental setup and the FF-WIOS2 evaluation board, shown in Figure 6, were used for electrical stimulation on the left V1 lobe of the animal subject. We observed the LFPs under 700 µA stimulus, 100 µA stimulus, and those that are spontaneous to compare the evoked activity. In Figure 15a, as expected, larger stimulation current induces larger LFP variations. Figure 15b shows the PSD results extracted from the averaged LFPs over 100 electrical stimulations. In a short delay after the stimulation, a significant increase and neuronal oscillations in PSD are observed. However, the stimuli at 100 µA only causes a slight increase in PSD as compared to LFP PSD under lower stimulation strength.

## 4. Discussion and Conclusion

We have presented a mm-sized free-floating wirelessly-powered implantable opto-electro stimulation device. It is efficiently powered through an optimized 3-coil inductive link, which also carriers user-defined stimulation parameters in downlink data transmission and SoC supply voltage information in uplink data transmission. The SoC circuit topology for each block is chosen for not only power/area efficiency but also design simplicity and reliability. The SCS method implemented in the SoC allows storage of wirelessly delivered energy in the storage capacitor, *C*_S_, and generation of large instantaneous power needed to drive the selected µLED and generate flashes that pass the minimum threshold of optogenetic stimulation in the neural tissue despite the weak inductive coupling of a mm-sized device. The SCS method is also capable of safe electrical stimulation with a combination of active and passive charge balancing. Both optical and electrical stimulation functions have been successfully verified in vivo by analyzing the evoked LFPs and immunohistochemical analysis.

The demand for the amount of power delivered to the selected µLED depends on whether the intensity of the resulting flash of light within the tissue can surpass the optogenetic neuromodulation threshold of 1mW/mm^2^ [29]. This would require sufficient light generated at the light source (µLED) and low-loss delivery of that light to the neural tissue. The first factor has been addressed by the on-chip SCS architecture. The second factor is out of the scope of this particular paper, but it has been addressed in our earlier publications, such as [37]. To maintain the normal operation of the SCS, the power delivered to the SoC should be at a level that is just enough for fully charging the storage capacitor, *C*_S_, before the next cycle of stimulation at its highest rate, while preventing power dissipation beyond safe limits due to extra electromagnetic absorption and heat generation.

Table 2 benchmarks the FF-WIOS2 device against state-of-art stimulation devices in the literature. In [21] and [22], they show distinct advantages in device size and weight over other designs listed in Table 2. However, they can only support one stimulation type with a single channel. Even though the device proposed in [26] has both optical and electrical stimulation functions, it is too bulky to be implanted. In [27], both optical and electrical stimulation functions were successfully implemented in a compact device at low channel count. Compared to the state-of-the-arts, the proposed FF-WIOS2 device benefits from (1) compact size and light weight, (2) high-level integration of 16-ch optical and 4-ch electrical stimulation, (3) flexibility for users to specify the stimulation type and patterns without the need for explantation, (4) capable of proving high instantaneous stimulation current, and (5) performance evaluation in vivo.

We are now working towards building the miniaturized and biocompatible FF-WIOS2 device. We have developed a dual-band EnerCage-HC system capable of wirelessly powering mm-sized implantable devices in a rodent standard-sized homecage [38]. We plan to test the miniaturized FF-WIOS2 device in vivo first on anesthetized rats and then on freely behaving subjects within the dual-band EnerCage-HC system environment [38]. Given that the aqueous and tissue medium will change the resonance frequency of the implanted *L*_Res_ and *L*_Rx_, we will integrate an adaptive capacitor tuning circuit in the next generation of the SoC design. The adaptive capacitor tuner, referring to our previous design in [39], can compensate for the resonance capacitance variation, eventually ensuring the inductive link at resonance. In addition to the functional test of the miniaturized FF-WIOS2 device, the device stability and biocompatibility after being fully implanted in the animal subject will also be evaluated.

## Figures and Tables

**Table 1:** FF-WIOS2 SoC Measured Specifications.

**Overall System**			
Chip area		1 mm^2^	
Power consumption w/o stimulation		320 µW	
Voltage doubler efficiency		43%	
**Switched-Capacitor Based Stimulation**			
Target voltage		5 V	
Charging efficiency		37%	
Charging time		50 ms	
*C*_S_/*C*_IN_/*C*_L_		10 µF or 1 µF / 10 µF / 10 µF	
Optical stim. efficiency		62.5%	
**Stimulation**	**Electrical**		**Optical**
Frequency	20–200 Hz, 2 bits		1–10 Hz, 2 bits
Pulse width	50–350 µs, 2 bits		1.6–6.4 ms, 2 bits
Current limiter	100–700 µA, 3 bits		1.7–12 mA, 3 bits
Light intensity	NA		1.5–11.8 mW/mm^2^
**Forward and Back Telemetry**			
Data bits		20 bits	
Pre/post-amble bits		10 bits	
PPM data rate		50 kbps	
LSK data rate		160–1280 Hz, 2 bits	
Back telemetry pulse width		1 or 2 µs, 1 bit	

**Table 2:** FF-WIOS2 SoC Measured Specifications.

Publications		[21]	[22]	[23]	[26]	[27]	This Work
Technology		0.18-µm RF	0.35-µm CMOS	COTS	COTS	0.18-µm HV BCD	**0.35-µm CMOS**
Wireless power transmission		Inductive link, 1.18 GHz	Photovoltaic	Inductive link, 13.56 MHz	Inductive link, 1.5 MHz	Ultrasound, 1.314 MHz	**Inductive link, 60 MHz**
Device size (mm^3^)		0.009	1.3 × 1.3 × (0.6–10)	9.8 mm diameter	30 × 25 × 10	2 × 3 × 6.5	**2.5 × 2.5 × 1.5**
Device weight		< 1 mg	2.3 mg	30 mg	7 g	78 mg	**15 mg**
Optical stim.	Channel #	-	1	1	8	1	**16**
	Current	-	5 mA	20 mA	Up to 25 mA	22 µA-5 mA	**1.7 mA–12 mA**
	Light intensity	-	Up to 15 mW/mm^2^	1–50 mW/mm^2^	-	1.4–23 mW/mm^2^	**1.5–11.8** **mW/mm^2^**
Electrical stim.	Channel #	1	-	-	16	4	**4**
	Current	38 µA	-	-	50 µA–10 mA	22 µA–5 mA	**100 µA–700 µA**
	Charge balance	No	-	-	Passive	Passive	**Active + Passive**
In vivo experiments		Yes	No	Yes	Yes	Yes	**Yes**
